# Cardiac autonomic imbalance by social stress in rodents: understanding putative biomarkers

**DOI:** 10.3389/fpsyg.2014.00950

**Published:** 2014-08-26

**Authors:** Susan K. Wood

**Affiliations:** Department of Pharmacology, Physiology and Neuroscience, School of Medicine, University of South CarolinaColumbia, SC, USA

**Keywords:** social defeat, corticotropin releasing factor, neuropeptide Y, inflammation, post traumatic stress disorder, anxiety, depression, heart rate variability

## Abstract

Exposure to stress or traumatic events can lead to the development of depression and anxiety disorders. In addition to the debilitating consequences on mental health, patients with psychiatric disorders also suffer from autonomic imbalance, making them susceptible to a variety of medical disorders. Emerging evidence utilizing spectral analysis of heart rate variability (HRV), a reliable non-invasive measure of cardiovascular autonomic regulation, indicates that patients with depression and various anxiety disorders (i.e., panic, social, generalized anxiety disorders, and post traumatic stress disorder) are characterized by decreased HRV. Social stressors in rodents are ethologically relevant experimental stressors that recapitulate many of the dysfunctional behavioral and physiological changes that occur in psychological disorders. In this review, evidence from clinical studies and preclinical stress models identify putative biomarkers capable of precipitating the comorbidity between disorders of the mind and autonomic dysfunction. Specifically, the role of corticotropin releasing factor, neuropeptide Y and inflammation are investigated. The impetus for this review is to highlight stress-related biomarkers that may prove critical in the development of autonomic imbalance in stress -related psychiatric disorders.

Stress that stems from one’s social environment is the most common form of stress encountered by people and is perceived as more intense than other types of stressors (Almeida, 2005). Socially stressful events are well recognized as contributing to the pathogenesis of depression, anxiety and posttraumatic stress disorder (PTSD; Kessler, 1997; Kendler et al., 1999; Javidi and Yadollahie, 2012). In addition to the debilitating consequences these psychiatric disorders have on mental health, they are also strongly associated with cardiovascular disease (CVD). As a result, psychiatric disorders significantly increase the risk of cardiac morbidity and mortality (Anda et al., 1993; Barefoot et al., 1996; Penninx et al., 2001; Rugulies, 2002; Surtees et al., 2008). Despite the clinical association between stress-related psychiatric disorders and CVD, little is understood regarding the pathophysiology or biomarkers underlying these comorbid disorders.

## AUTONOMIC IMBALANCE IN PATIENTS WITH PSYCHIATRIC DISORDERS: RELEVANCE TO HEART DISEASE

One physiological change observed in patients with psychiatric disorders thought to contribute to increased CVD risk is dysfunction within the autonomic nervous system as evidenced by changes in heart rate variability (HRV). HRV reveals beat-to-beat changes in heart rate measured by the electrocardiogram. Variability within the heart rate is regulated by the sympathetic nerves, which accelerate heart rate and the parasympathetic (vagus) nerve, which slows it. Therefore, HRV provides a non-invasive measure of the balance between the sympathetic and parasympathetic nervous system. Healthy cardiac activity is characterized by a high degree of parasympathetic input and thus increased variability, protecting the heart against adverse cardiac events such as heart failure and myocardial infarction (Bigger et al., 1988; Hughes and Stoney, 2000; Carney et al., 2001). Alternatively, reductions in HRV that reflect increased sympathetic tone increase the risk of cardiac arrhythmias and sudden cardiac death (Verrier and Lown, 1982; La Rovere et al., 2003). Spectral analysis of HRV has revealed that increased low frequency (LF) measure of HRV and decreased high frequency (HF) reflect enhanced sympathetic and decreased parasympathetic activity, resulting in a marked increase in the LF/HF ratio (Pagani et al., 1986; Berntson et al., 1997; Ramaekers et al., 2002). Importantly, patients suffering from stress-related psychiatric disorders also exhibit autonomic disturbances. Major depression, generalized anxiety disorders and PTSD have all been associated with reductions in HRV (Udupa et al., 2007; Kemp et al., 2010; Pittig et al., 2013) and suffering from two of these disorders concurrently is reported to produce a further reduction in HRV (Chang et al., 2013; Minassian et al., 2014). A shift in HRV of this manner predicts life-threatening cardiac arrhythmias (Huikuri et al., 2009) and in patients lacking preexisting CVD, stress-related disorders such as depression and anxiety are associated with significantly increased risk of adverse cardiac events (Barefoot et al., 1996; Carney et al., 2001). While this association is clearly documented, the molecular systems or biomarkers capable of generating both stress-related psychiatric disorders and CVD are not defined, making treatment challenging. Preclinical models of social stress have provided invaluable clues as to which biological markers may be involved in the pathogenesis of these comorbid diseases and will be the focus of this review.

## SOCIAL STRESS-INDUCED DYSFUNCTION IN RODENTS: RELEVANCE TO HUMAN PSYCHIATRIC AND CVD COMORBIDITY

Stress is a common risk factor for both CVD and psychiatric disorders. Therefore, identifying stress-sensitive systems that mediate cardiovascular and behavioral/emotional responses could shed light on the shared pathophysiology that links these comorbid disorders. Two reliable ethologically relevant animal model of social stress have proven particularly useful for studying this link. One is the resident-intruder paradigm of social defeat (Miczek, 1979; Sgoifo et al., 2014). This model involves subjecting a male rat (intruder) to aggressive threats from a larger, unfamiliar male rat (resident) by placing it in the resident’s home cage for a short period of time. In the acute sense (minutes to hours) social defeat produces robust sympathetic activation eliciting 30 times the number of arrhythmias (ventricular premature beats) as compared to other non-social stressors such as restraint or foot shock (Sgoifo et al., 1999). Social defeat also produces vagal withdrawal, tachycardia, hypertension, hyperthermia, elevated plasma catecholamines, and increased activation of the hypothalamic-pituitary-adrenal axis (Tornatzky and Miczek, 1993, 1994; Sgoifo et al., 1999; Bhatnagar et al., 2006; Wood et al., 2010). Another valid model is social isolation in the socially monogamous prairie voles. This model involves separating a prairie vole from its opposite-sex partner and is well characterized as producing long-term cardiovascular dysfunction within 2–4 weeks. Under resting conditions, isolated prairie voles exhibit elevated heart rate, decreased HRV (Grippo et al., 2007), and a pronounced increase in circulating corticosterone and adrenal weights (Bosch et al., 2009). The robust activation of the sympathetic nervous system and the HPA axis during social defeat and social isolation are similar to those observed in humans in response to the Trier Social Stress Test, an experimental model of social stress in humans (Hellhammer and Schubert, 2012).

Acute stress responses are adaptive in helping the individual cope with the stressor, however, if unabated in the face of chronic stress this can lead to pathological changes. Therefore, social defeat in rodents and social isolation in prairie voles have behavioral and physiological consequences that are relevant to human pathologies. Cardiovascular telemetry allows for detailed 24 h cardiac monitoring in unrestrained animals and has been critical in establishing cardiovascular repercussions to stress, allowing for direct comparison to humans. From a physiological perspective, social defeat renders rats with disruption of the circadian rhythm for heart rate and core temperature (Tornatzky and Miczek, 1993; Sgoifo et al., 1999, 2002; Meerlo et al., 2002) and maladaptive cardiac hypertrophy (Gelsema et al., 1994). Seven brief exposures to social defeat increased the LF/HF ratio at rest, indicating a shift of sympathovagal balance toward a relative prevalence of sympathetic modulation (Wood et al., 2012). These findings were extended in a recent report where as few as four brief exposures to social defeat produced chronic reductions in both HRV and cardiac baroreflex sensitivity, reflecting a shift toward sympathetic predominance (Sevoz-Couche et al., 2013) which have been shown to be predictive of the occurrence of life threatening arrhythmias (Billman et al., 1982; La Rovere et al., 2003). Similarly, in prairie voles social isolation reduces resting HRV, increases cardiac weight and increases susceptibility to forced swim-induced arrhythmias (Grippo et al., 2007, 2012). Dysfunction within both the HPA axis and the immune system of socially stressed animals are also reported as persistent outcomes and mimic maladaptive changes seen in people with psychiatric diseases (Stefanski, 1998; Buwalda et al., 1999; Bhatnagar and Vining, 2003; Wood et al., 2010). Relevant long lasting behavioral consequences include decreased motivation, increased behavioral despair, anhedonia, anxiety-like behaviors, and decreased social interactions (Von Frijtag et al., 2000; Rygula et al., 2005; Grippo et al., 2007; Becker et al., 2008; Wohleb et al., 2011; Patki et al., 2013). Taken together, these studies provide evidence that repeated social defeat in rodents or chronic social isolation in prairie voles possesses the unique ability to recapitulate the behavioral and physiological changes associated with depressive and anxiety disorders. Taken together, these social stressors represent relevant models to study the comorbidity between psychiatric disorders and CVD, shedding light on mechanisms and biomarkers that may lead to increased susceptibility to psychiatric disorders with comorbid autonomic dysfunction. The following sections of this review report evidence from clinical and preclinical social stress studies highlighting putative biomarkers that should be further evaluated for their role in the pathogenesis of these comorbid conditions.

## PUTATIVE BIOMARKER INVOLVED IN PSYCHIATRIC-CVD COMORBIDITY: CORTICOTROPIN-RELEASING FACTOR

There are several stress-sensitive biological molecules that have both pro-depressive or anxiogenic effects and impact the autonomic nervous system. As such, these molecules are classified as putative biomarkers driving the comorbidity between psychiatric disorders and autonomic dysfunction. One potential biomarker is corticotropin-releasing factor (CRF). This neuropeptide is considered the “hallmark” of the stress response (Vale et al., 1981). In stress-sensitive regions of the brain such as the amygdala, locus coeruleus (LC) and dorsal raphe CRF receptor activation is involved in stress-related emotionality and produces behavioral features of the stress response (Heinrichs et al., 1992; Hammack et al., 2003; Ayala et al., 2004; Wood and Woods, 2007; Dunn and Swiergiel, 2008; Valentino et al., 2009). Central administration of CRF in rats also activates the sympathetic nervous system, resulting in a marked pressor response and tachycardia (Briscoe et al., 2000; Nijsen et al., 2000). CRF’s influence on the sympathovagal balance also extends into the periphery; intravenous infusion of CRF transiently increased the LF/HF ratio and tachycardia (Arlt et al., 2003). Given CRF’s pervasive influence, it plays a central role in the behavioral, neuroendocrine and cardiovascular limbs of the stress response.

Like many elements of the stress response CRF is capable of promoting healthy adaptation to stress (Vale et al., 1981), but when unabated it can lead to pathology. For example, social defeat and social isolation impacts CRF levels as well as CRF_1_ receptor distribution and quantity in brain and pituitary (Wood et al., 2009, 2010, 2013; Pournajafi-Nazarloo et al., 2011; Chaijale et al., 2013). Moreover, transgenic mice engineered to over-express CRF in the brain are disposed to exhibiting a depressive- and anxiety-like phenotype as well as decreased HRV (Dirks et al., 2002; Vicentini et al., 2009; Bangasser et al., 2013). Overproduction of central CRF as evidenced by increased CRF has been identified in CSF of patients with depressive disorders and anxiety disorders such as PTSD (Nemeroff et al., 1984; Bremner et al., 1997; Baker et al., 1999). In post mortem depressed patients, specific changes in CRF within brain regions implicated in psychiatric disorders are also documented. For example, increased CRF protein levels have been documented in the LC and the paraventricular nucleus of the hypothalamus (Raadsheer et al., 1994; Austin et al., 2003; Bissette et al., 2003). Furthermore, CRF receptor mRNA down-regulation was reported in the frontal cortex of depressed patients and was thought to be a secondary consequence of exaggerated CRF release (Merali et al., 2004). Due to the convincing link between CRF dysfunction and psychiatric disorders, clinical trials have evaluated the therapeutic efficacy of CRF_1_ antagonists. While the results have been equivocal, there is evidence to support their use as a promising new pharmacotherapy for anxiety and depression (Holsboer and Ising, 2008). The ambiguous nature of clinical results evaluating CRF_1_ antagonists is suggested to be, in part, due to testing ineffective doses and the wrong patient population (Belzung, 2014). Preclinical studies have suggested that disorders with a high contribution of stress in the etiology, such as PTSD and stress-induced depression, may benefit from CRF antagonists (Wood et al., 2012; Philbert et al., 2013) however, individuals with stress-induced disorders were not tested (Belzung, 2014). For example, CRF antagonist treatment blocked depressive-like behavior following social isolation in prairie voles (Bosch et al., 2009). Furthermore, our recent studies revealed that social defeat-induced depressive-like behaviors, HPA dysfunction and decreased HRV was blocked by a CRF_1_ antagonist during stress (Wood et al., 2010, 2012). Therefore, converging lines of evidence underscore the role of CRF in the development of stress-induced comorbidity between depression or anxiety and CVD.

## PUTATIVE BIOMARKER INVOLVED IN PSYCHIATRIC-CVD COMORBIDITY: NEUROPEPTIDE Y

Neuropeptide Y (NPY) is yet another neuroendocrine peptide that has demonstrated central control over both behavioral and cardiovascular responses to stress. NPY is widely distributed in the brain and expressed in regions implicated in psychiatric disorders. NPY is often co-expressed with the neuropeptide CRF and as such, it is poised to impact central regulation of stress-related behavior, neuroendocrine and cardiovascular responses. For example, the LC (Makino et al., 2000), the amygdala (Adrian et al., 1983), and the PVN (Baker and Herkenham, 1995) all highly express both neuropeptides and NPY is reported to oppose the effects of CRF (Heilig et al., 1994; Britton et al., 2000). One such example occurs in the LC, where CRF serves as an excitatory neurotransmitter (Valentino et al., 1983) and NPY reduces the firing of LC noradrenergic neurons (Illes et al., 1993). As a result, central administration of NPY decreases NE overflow by acting on Y_1_ receptors (Hastings et al., 2004). Because evidence of elevated LC activity has been linked to depression and PTSD (Wong et al., 2000; Geracioti et al., 2001) this NPY-induced brake on LC over activation may therefore promote stress resilience. The anti-stress effect of NPY is not unique to the LC; decreased levels of NPY were observed in the amygdala, hippocampus and periaqueductal gray of rats that were vulnerable to predator-scent stress versus the resilient phenotype (Cohen et al., 2012). Furthermore, social defeat exposure decreases NPY and NPY receptor mRNA in the hippocampus and hypothalamus (Zambello et al., 2010). Elevated NPY levels have also been associated with resistance to an anxious phenotype; in rats characterized as exhibiting high or low levels of anxiety, NPY mRNA in the amygdala was negatively correlated with anxious behavior (Primeaux et al., 2006). Moreover, central administration of exogenous NPY has demonstrated anxiolytic properties in rodents and is capable of inhibiting the anxiogenic effects of CRF (Britton et al., 1997; Ehlers et al., 1997; Primeaux et al., 2005). Importantly, these preclinical data are relevant to findings in humans; deficiencies within the central NPY system have been demonstrated in patients with major depression (Widerlov et al., 1988). Combat-exposed individuals with PTSD also have significantly lower levels of NPY in CSF (Rasmusson et al., 2000; Sah et al., 2014) and NPY levels recover following remission (Yehuda et al., 2006). Along these lines, high levels of NPY were observed in highly resilient special operations soldiers (Morgan et al., 2000). Therefore, clinical and preclinical data point toward increased NPY promoting resilience, while reduced NPY in the brain is related to psychiatric disorders.

In addition to its cognitive effects, NPY has prominent cardiovascular impact. Like NPY’s effect on anxiety, its hemodynamic effects are also in contrast to CRF; central administration of NPY lowers blood pressure and heart rate at rest and in response to social defeat in rats (Klemfuss et al., 1998). The marked depressor and bradycardic actions of NPY occur at doses that have potent anxiolytic properties (Britton et al., 1997; Klemfuss et al., 1998). However, while the central actions of NPY may be cardioprotective, peripheral NPY infusion in rodent and in man acts as a potent vasoconstrictor (Pernow et al., 1987). NPY is co-released with NE during conditions of high-intensity sympathetic nerve activity and studies in mice lacking the NPY_1_ receptor revealed that NPY serves to potentiate NE-evoked vasoconstriction (Lundberg et al., 1986; Pedrazzini et al., 1998). As such, peripheral NPY is associated with CVD such as heart failure, hypertension and myocardial ischemia (Zukowska-Grojec et al., 1996). Nonetheless, therapies that increase NPY selectively in the brain may prove effective in treating anxiety or depressive disorders and decrease cardiovascular risk by reducing sympathetic activity. In rodents, the single prolonged stress PTSD model produces many behavioral and biochemical features of PTSD (Liberzon et al., 1997) and in a recent study, intranasal NPY effectively blocked or reversed many of the consequences of this stressor (Serova et al., 2013, 2014). Several lines of evidence link NPY with the psychobiology of resilience to psychiatric disorders and CVD comorbidity. Therefore, investigating the efficacy of intranasal NPY for PTSD and depression as well as mitigating CVD risk in these patients will be critical.

## PUTATIVE BIOMARKER INVOLVED IN PSYCHIATRIC-CVD COMORBIDITY: INFLAMMATORY CYTOKINES

Proinflammatory cytokines are another such mediator that can be persistently up regulated as a result of stress in vulnerable individuals and has roots in the pathogenesis of both psychiatric disorders and CVD (Black and Garbutt, 2002). In fact, inflammation has long been recognized as contributing to CVD and recently, converging evidence implicates inflammatory factors in the pathogenesis of depression and anxiety disorders. Patients suffering from depression exhibit increased levels of the proinflammatory cytokine interleukin-6 (IL-6) while at rest and exhibit greater social stress-induced IL-6 levels, which are normalized following antidepressant therapy (Frommberger et al., 1997; Pace et al., 2006; Fagundes et al., 2013). Furthermore, Infliximab, a monoclonal antibody against tumor necrosis factor-α (TNF-α), exhibited antidepressant efficacy in a subset of patients characterized by elevated plasma cytokines (Raison et al., 2013). Elevated inflammation has also been identified in patients with various anxiety disorders, including PTSD (Vogelzangs et al., 2013; Newton et al., 2014). Interestingly, NPY has an inhibitory influence on neuroinflammation, further supporting the role of NPY in resilience (detailed review in Malva et al., 2012). Preclinical data also support the role of inflammation in depressive-like behaviors; IL-6 knockout mice demonstrate decreased depressive-like behaviors (Chourbaji et al., 2006). In addition to the unique capability of social defeat to produce an anxiety- and depressive-like phenotype with cardiovascular alterations as discussed above, it has been reported to produce persistent increases in the proinflammatory markers IL-6 and TNFα (Kinsey et al., 2008). Furthermore, microglia isolated from the brains of socially defeated mice produced markedly higher levels of the proinflammatory molecules IL-6, TNF-α, and monocyte chemoattractant protein-1 in response to the endotoxin lipopolysaccharide compared with controls (Wohleb et al., 2011). Importantly, antidepressants attenuate inflammation-induced brain cytokine production, as well as depressive-like symptoms, further supporting a role for neuroinflammation in depressive disorders in humans (Castanon et al., 2001; Yirmiya et al., 2001).

Proinflammatory cytokines also possess potent cardiovascular effects. For example, both central and peripheral infusion of the proinflammatory cytokine interleukin-1β induces a pressor response and tachycardia in rats (Kannan et al., 1996). In humans, cytokines are also linked to the autonomic nervous system, as reductions in HRV are associated with higher concentrations of IL-6 and TNF-α (Gonzalez-Clemente et al., 2007; von Kanel et al., 2008). A growing, yet still poorly understood body of evidence points toward neuroinflammation in the psychopathology of stress-related disorders and must be further characterized for the impact anti-inflammatory therapies may have on mitigating CVD risk in these patients (Dantzer et al., 2008; Maes et al., 2009; Rawdin et al., 2013).

There is a large body of evidence to suggest that psychosocial stress plays a prominent role in the etiology and progression of certain CVDs and psychiatric disorders. As such, there is a strong association between psychiatric disorders and increased cardiac morbidity and mortality. This review highlights proinflammatory cytokines, CRF, and NPY systems amongst those capable of generating both depressive or anxiety-like behaviors and reductions in HRV, thereby increasing CVD risk. Targeting these systems in preclinical social stress models and in psychiatric disorder patients with evidence of decreased HRV may prove beneficial and warrant further evaluation.

## Conflict of Interest Statement

The author declares that the research was conducted in the absence of any commercial or financial relationships that could be construed as a potential conflict of interest.
